# Gastric *Helicobacter* species associated with dogs, cats and pigs: significance for public and animal health

**DOI:** 10.1186/s13567-022-01059-4

**Published:** 2022-06-13

**Authors:** Emily Taillieu, Koen Chiers, Irina Amorim, Fátima Gärtner, Dominiek Maes, Christophe Van Steenkiste, Freddy Haesebrouck

**Affiliations:** 1 Department of Pathobiology, Pharmacology and Zoological Medicine, Faculty of Veterinary Medicine, Ghent University, Merelbeke, Belgium; 2 Instituto de Investigação E Inovação Em Saúde (i3S), Universidade Do Porto, Porto, Portugal; 3 Institute of Pathology and Molecular Immunology, University of Porto (IPATIMUP), Porto, Portugal; 4 School of Medicine and Biomedical Sciences, Porto University, Porto, Portugal; 5 Department of Internal Medicine, Reproduction and Population Medicine, Faculty of Veterinary Medicine, Ghent University, Merelbeke, Belgium; 6 Department of Gastroenterology and Hepatology, University Hospital Antwerp, Antwerp University, Edegem, Belgium; 7 Department of Gastroenterology and Hepatology, General Hospital Maria Middelares, Ghent, Belgium

**Keywords:** Gastric *Helicobacter* species, non-*Helicobacter pylori Helicobacter* species, gastric disease, zoonosis, dog, cat, pig, human

## Abstract

This article focuses on the pathogenic significance of *Helicobacter* species naturally colonizing the stomach of dogs, cats and pigs. These gastric “non-*Helicobacter* (*H.*) *pylori Helicobacter* species” (NHPH) are less well-known than the human adapted *H. pylori*. *Helicobacter suis* has been associated with gastritis and decreased daily weight gain in pigs. Several studies also attribute a role to this pathogen in the development of hyperkeratosis and ulceration of the non-glandular stratified squamous epithelium of the *pars oesophagea* of the porcine stomach*.* The stomach of dogs and cats can be colonized by several *Helicobacter* species but their pathogenic significance for these animals is probably low. *Helicobacter suis* as well as several canine and feline gastric *Helicobacter* species may also infect humans, resulting in gastritis, peptic and duodenal ulcers, and low-grade mucosa-associated lymphoid tissue lymphoma. These agents may be transmitted to humans most likely through direct or indirect contact with dogs, cats and pigs. Additional possible transmission routes include consumption of water and, for *H. suis*, also consumption of contaminated pork. It has been described that standard *H. pylori* eradication therapy is usually also effective to eradicate the NHPH in human patients, although acquired antimicrobial resistance may occasionally occur and porcine *H. suis* strains are intrinsically less susceptible to aminopenicillins than non-human primate *H. suis* strains and other gastric *Helicobacter* species. Virulence factors of *H. suis* and the canine and feline gastric *Helicobacter* species include urease activity, motility, chemotaxis, adhesins and gamma-glutamyl transpeptidase. These NHPH, however, lack orthologs of cytotoxin-associated gene pathogenicity island and vacuolating cytotoxin A, which are major virulence factors in *H. pylori.* It can be concluded that besides *H. pylori*, gastric *Helicobacter* species associated with dogs, cats and pigs are also clinically relevant in humans. Although recent research has provided better insights regarding pathogenic mechanisms and treatment strategies, a lot remains to be investigated, including true prevalence rates, exact modes of transmission and molecular pathways underlying disease development and progression.

## Introduction

The stomach has long been considered a sterile and hostile environment for bacteria because of the extremely acidic conditions. However, the opposite is true. Among other resident microbes of the stomach, various gastric *Helicobacter* species are able to colonize the stomach of animals and humans and may cause gastric disease [1–3]. *Helicobacter* (*H.*) *pylori* is by far the best known and best studied gastric *Helicobacter* species with a global prevalence in humans of more than 50%, where this prevalence is generally higher in low-income countries compared to developed countries [4]. *Helicobacter pylori* infection has been associated with severe gastric disease in humans as it may cause gastritis, peptic ulcer disease, gastric carcinoma and low-grade gastric B cell mucosa-associated lymphoid tissue (MALT) lymphoma [5, 6]. This bacterium was isolated from a human gastric biopsy for the first time in 1982 by Marshall and Warren [7]. Since then, many other gastric *Helicobacter* species have been identified in human patients’ stomachs, admittedly at a much lower prevalence [8–10]. These gastric non-*Helicobacter pylori Helicobacter* species (NHPH) naturally reside in animal hosts, including dogs, cats and pigs. To date, the *Helicobacter* genus includes 48 distinct, validly published species, among which 17 gastric and 31 enterohepatic NHPH [11, 12]. In recent years, increasingly more insights have been gained regarding the pathogenic significance and zoonotic potential of the hitherto identified gastric NHPH, however, a lot remains to be investigated.

This review aims to provide an overview of the existing evidence concerning the pathogenic significance of dog-, cat- and pig-associated gastric NHPH in their respective animal hosts and in human patients.

## Taxonomy of gastric non-*Helicobacter pylori Helicobacter* species

Only recently a consensus has been reached on the complex nomenclature of the gastric NHPH, a still expanding group of helicobacters. First, the name “*Gastrospirillum hominis*” was proposed in 1989 after the first discovery of Gram-negative, long, helical-shaped bacteria, morphologically distinct from the curve-shaped *H. pylori*, residing in the gastric mucosa of patients with gastrointestinal symptoms [13]. Based on *16S rRNA* gene sequencing, it was discovered that these bacteria actually belonged to the *Helicobacter* genus and so, the NHPH were renamed to “*H. heilmannii*”, after Konrad Heilmann who described for the first time a large case study of NHPH infections in humans [9, 14, 15]. Further genetic analysis showed significant differences in the *16S rRNA* gene nucleotide sequence among different species within this group of gastric NHPH, which led to a subclassification into “*H. heilmannii*” type 1 and “*H. heilmannii*” type 2 [9, 16, 17].

In 1990, another “*Gastrospirillum*” species was discovered residing in the gastric mucosa of pigs, named “*Gastrospirillum suis*” [18, 19]. Through sequencing of the *16 rRNA* gene, fluorescent in situ hybridization and electron microscopy studies, it was proven that “*Gastrospirillum suis*” belonged to the *Helicobacter* genus and sequencing of the *urease A* and *B* (*ureAB*) genes proved that it was identical to “*H. heilmannii*” type 1. Therefore, the name “*Candidatus* Helicobacter suis” was proposed and the bacterium was designated a new taxon [17, 20, 21]. The term “*Candidatus*” indicates that, at that time, the agent could not yet be isolated and cultivated in vitro [22]. Acquirement of the first in vitro isolates of this bacterium in 2006 led to its characterization as *H. suis* [17].

“*Helicobacter heilmannii*” type 2 on the other hand represents several dog- and cat-associated gastric NHPH which have been proven to be genetically distinct upon isolation and characterization. These include *H. felis* [23], *H. bizzozeronii* [24], *H. salomonis* [25], *H. cynogastricus* [26], *H. baculiformis* [27], *H. heilmannii* sensu stricto (*H. heilmannii* s.s.) [21, 28] and *H. ailurogastricus* [29].

To avoid confusion concerning the term *H. heilmannii*, it has been proposed to use the terms *H. heilmannii* sensu lato (*H. heilmannii* s.l.) and *H. heilmannii* s.s. [30]. The term *H. heilmannii* s.l. is then used to refer to all gastric NHPH, which is useful when only results of histopathology or electron microscopy or crude taxonomic data are available, whereas the term *H. heilmannii* s.s. or another specific species name of a gastric NHPH refers to a single fully characterized NHPH.

Sequencing of the *16S rRNA* or *23S rRNA* genes allows differentiation between the pig-associated *H. suis* and the group of dog- and cat-associated gastric NHPH [16]. However, it does not allow to differentiate between the different dog- and cat-associated gastric NHPH [26, 27]. To achieve this anyway, sequencing of the (partial) *ureAB* genes is the preferred method since the sequences of these genes are available for all gastric NHPH [9, 21]. Moreover, sequencing of the *ureAB* genes does not allow differentiation between *H. heilmannii* s.s. and its closest relative *H. ailurogastricus* [29, 31]. These two species can be distinguished through genome sequencing based approaches [29] or sequencing of the *IceA* gene and *LpsA* gene [32]. Whole cell protein profiling is another method to distinguish the gastric NHPH, however this method requires pure in vitro cultures [30]. More recently, MALDI-TOF mass spectrometry has been shown to allow differentiation between gastric *Helicobacter* species cultured in vitro [33]. Given the fastidious nature of the gastric NHPH, in vitro isolation and cultivation is complicated, as is characterization of potential new species detected in human and animal stomachs [9, 30]. An overview of the dog-, cat- and pig-associated gastric NHPH and their corresponding phenotypic characteristics is presented in Table 1.

**Table 1:** **Phenotypic characteristics of dog-, cat- and pig-associated gastric non-*Helicobacter pylori Helicobacter*** **species**

Characteristic	*H. suis*	*H. felis*	*H. bizzozeronii*	*H. salomonis*	*H. heilmannii* s.s.	*H. ailurogastricus*	*H. baculiformis*	*H. cynogastricus*
Length (µm)	2.3–6.7	5–7.5	5–10	5–7	3–6.5	3–5.5	10	10–18
Width (µm)	0.9–1.2	0.4	0.3	0.8–1.2	0.6–0.7	0.5–0.7	1	0.8–1
Periplasmic fibrils	−	+	−	−	−	−	+	+
Number of flagella per cell	4–10	14–20	10–20	10–23	4–10	6–8	11	6–12
Distribution of flagella	Bipolar	Bipolar	Bipolar	Bipolar	Bipolar	Bipolar	Bipolar	Bipolar
Flagellar sheath	+	+	+	+	+	+	+	+
Urease	+	+	+	+	+	+	+	+
Catalase	+	+	+	+	+	+	+	+
Oxidase	+	+	+	+	+	+	+	+
Nitrate reduction	−	+	+	+	+	+	+	+
Alkaline phosphatase	+	+	+	+	−	+	+	+
Indoxyl acetate hydrolysis	−	−	+	+	−	−	−	−
γ-glutamyl transpeptidase	+	+	+	+	+	+	+	+
Growth at 25 °C	−	−	−	−	−	−	−	−
Growth at 30 °C	ND	ND	ND	ND	ND	ND	ND	+
Growth at 37 °C	+	+	+	+	+	+	+	+
Growth at 42 °C	−	+	+	−	−	−	−	−
Growth on 1% glycine	−	−	−	−	−	ND	−	−
Growth on 1% ox bile	−	ND	−	−	−	ND	−	−
Growth on 1.5% NaCl	−	−	−	−	−	ND	−	−
Type strain	HS1^T^ (= LMG 23995^ T^ = DSM 19 735^T^)	CS1 (= ATCC 49179)	CCUG 35 045 and CCUG 35 046	CCUG 37 845 (= Inkinen)	ASB1^T^ (= DSM 23 983^ T^ = LMG 26 292^T^)	ASB7^T^ (= DSM 100 489^ T^ = LMG 28 648^T^)	M50^T^ (= LMG 23 839^ T^ = CCUG 53 816^T^)	JKM4^T^ (= LMG 23 188^T^)
Type strain isolated from	pig	cat	dog	dog	cat	cat	cat	dog

Data were obtained from Baele et al*.* (2008) [17], Paster et al. (1991) [23], Hänninen et al. (1996) [24], Jalava et al. (1997) [25], Van den Bulck et al. (2006) [26], Baele et al. (2008) [27], Smet et al. (2012) [28], Joosten et al. (2016) [29], Bento-Miranda et al. (2014) [3] and Haesebrouck et al. (2009) [9].

+ , positive;  − , negative; ND, not determined.

## Pathogenic significance, prevalence and transmission of *Helicobacter suis* in pigs

**Figure 1:**
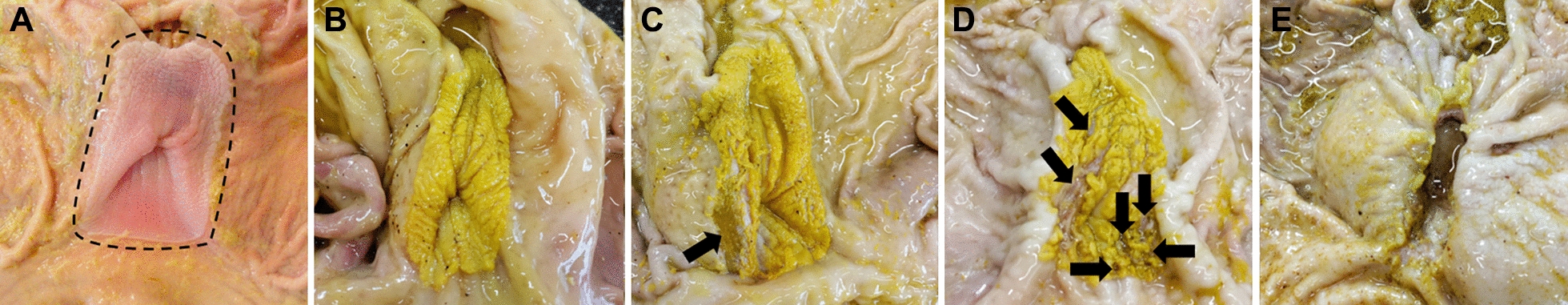
**Ulcer formation at the level of the** ***pars oesophagea*** **in slaughter pigs**. **A** normal mucosa; the *pars oesophagea* is delineated by a black dotted line, **B** severe hyperkeratosis, **C** hyperkeratosis with few erosions (erosions are indicated by black arrows), **D** hyperkeratosis with several erosions (erosions are indicated by black arrows), **E** ulceration.

*Helicobacter suis* infection in pigs has been associated with decreased daily weight gain [34], gastritis [35–37] and ulceration of the non-glandular part of the stomach, the *pars oesophagea* [9, 38–41], therefore impacting animal health, welfare and production. Images of lesions associated with ulcer formation at the level of the *pars oesophagea* of the porcine stomach are presented in Figure 1. *Helicobacter suis* colonizes the fundic and pyloric gland zone of the stomach. During the more acute phase of the infection, this may result in decreased gastric acid secretion in this distal, glandular part of the stomach, hereby affecting the composition of the microbiota of the *pars oesophagea*. An important factor in this microbiota composition shift was discovered to be related with an increased colonization by *Fusobacterium* (*F.*) *gastrosuis* [42]. Production of epithelial cell death inducing metabolites by *F. gastrosuis* potentially plays a role in porcine gastric ulceration [43]. During the more chronic phase of infection, secretion of excessive amounts of gastric acid in the distal part of the stomach is induced, which may lead to increased contact of the *pars oesophagea* with hydrochloric acid. As this part of the porcine stomach is not protected by mucus, hyperkeratosis, erosion and ulcer formation is likely to occur [9, 44]. To confirm this hypothesis, experimental studies involving pigs infected with *H. suis* and *F. gastrosuis* should be performed in order to find out which putative virulence factors are expressed and secreted and whether these are functional [43]. Furthermore, the etiology of ulceration of the *pars oesophagea* is most probably multifactorial and therefore, mere *H. suis* infection of the porcine stomach may not be sufficient to cause gastric ulcers [35]. Several predisposing factors associated with development of ulceration of the porcine stomach have been described, including nutritional, housing, management and environmental factors. Finely ground pelleted feed and disruptions in feed delivery are the two most important ones. Increased risk may also result from hot weather and outbreaks of acute infectious diseases [45]. Recently, Nunes et al*.* were able to detect *H. suis* DNA in the gastric mucosa of a wild boar, of which the stomach presented with macroscopic signs of antral inflammation [46].

Similar to what has been clearly demonstrated for *H. pylori*, some *H. suis* strains might be more pathogenic than others [47]. To this end, multilocus sequence typing (MLST) of *H. suis* strains has been executed, but a possible relationship between MLST type and virulence has not been demonstrated yet [48].

There are clear indications that *H. suis* originates from non-human primates, as it was discovered to colonize the stomach of rhesus and cynomolgus monkeys and a host jump from macaques to pigs would have happened between 100,000 and 15,000 years ago [8]. In general, host jumps like these are known to lead to an increase in disease severity, while coevolution between a bacterium and its natural host generally results in less severe pathogenicity. This accounts for *H. suis* too. Infections with *H. suis* are usually asymptomatic in non-human primates and cause gastric disease in pigs. Furthermore, pig domestication has significantly impacted the spread of *H. suis* within the pig population following the host jump [1].

Several independent studies conducted in different countries report different prevalence rates of *H. suis* colonizing the gastric mucosa of pigs at slaughter age, varying from about 10 to 60% or more, mainly depending on the geographic area, the diagnostic tools used and the management and hygiene standards applied [35–38, 41, 44]. The prevalence is found to be increasing with the pig’s age, being 2% in suckling piglets and 90% in adult pigs [49].

The low prevalence in suckling piglets is probably due to anti-microbial effects of the sow’s milk through protective antibodies and other antibacterial factors [50]. As this disappears after weaning, the prevalence of *H. suis* infections increases rapidly as regrouping of piglets favors the spread of the few *H. suis* infections already present before weaning to noninfected pigs [9]. Furthermore, the proportion of *Lactobacillus* populations in the upper gastrointestinal tract microbiota decreases upon weaning, possibly resulting in a higher risk of developing a *H. suis* infection. The decrease in the proportion of *Lactobacillus* populations attenuates the natural resistance against infection or colonization by pathogens. This is due to decreased competition for nutrients and epithelial binding sites, less production of antimicrobial factors such as lactic acid and bacteriocins and an increased pH in the gastrointestinal tract, creating a less hostile environment for bacteria [51, 52]. Therefore, the potential of *Lactobacillus* strains as probiotic bacteria that protect the gastric environment has been suggested, since they are abundantly present in porcine stomachs without ulceration of the *pars oesophagea* [52].

Since *H. suis* DNA has been detected in saliva but not in feces of naturally and experimentally infected pigs, transmission is suggested to occur via the oral-oral route or the gastric-oral route [9]. In one study *Helicobacter* DNA was detected by a genus specific PCR assay in saliva and fecal samples from pigs with gastric ulceration. All these samples reacted negative in a *H. suis* specific PCR assay, whereas *H. suis* DNA was frequently detected in gastric samples. As the researchers suggest, these findings may indicate that DNA from *Helicobacter* species other than *H. suis* was detected in saliva and feces from these pigs [53]*.* These might be enterohepatic *Helicobacter* species, since in previous studies several enterohepatic species, including *H. bilis* and *H. trogontum*, have been detected in and isolated from porcine fecal samples [54–56].

## Pathogenic significance, prevalence and transmission of dog- and cat-associated gastric non-*Helicobacter pylori Helicobacter* species in their natural hosts

The clinical significance of most gastric NHPH infections in cats and dogs remains controversial or yet to be explored. No conclusive evidence exists on the association between clinical signs, including chronic vomiting and inappetence, and the presence of gastric NHPH [57]. Concerning the more recently discovered NHPH *H. baculiformis*, *H. ailurogastricus* and *H. cynogastricus*, the clinical significance in their respective animal hosts as well as in humans remains unknown [26, 27, 29].

After investigating the clinical impact of the presence of gastric NHPH in the stomachs of cats showing clinical signs of gastrointestinal disease, Bridgeford et al. hypothesized that gastric NHPH infections in cats may be associated with feline gastric MALT lymphoma [58]. Scanziani et al. detected lymphoid follicular hyperplasia with mild pangastric mononuclear inflammation and eosinophilic infiltrates in cats naturally infected with *H. felis* [59]. Besides, correlations have been found between the degree of colonization by NHPH of the feline gastric mucosa and the extent of lymphocyte and plasma cell infiltration, the number of lymphoid follicles and the occurrence of fibrosis [60]. However, in cats diagnosed with *H. heilmannii* s.s. infection and infection with other, unclassified NHPH only minimal mononuclear inflammation was detected, as was also the case in uninfected cats. Indeed, different species and strains of NHPH may impact the severity of gastritis in cats, as well as the species and strain of the host, and the duration of infection. Furthermore, it has been suggested that induction of gastric inflammation may require factors other than *Helicobacter* infection alone [59]. In a more recent cohort study including 56 cats with and without chronic gastritis, gastric *Helicobacter* species specified as *H. heilmannii* s.s. were present in 28 cases (50%), with no evidence of significant associations with severity of gastric disease [61]. This supports evidence from another study in which *H. heilmannii* s.s. infection was frequently present in pet cats (38 of 49; 78%), although no significant correlation between the presence of infection and the degree of gastritis was found [62]. Similarly, Norris et al. found that in a cohort of healthy pet cats, infection with a gastric *Helicobacter* species closely related to *H. felis* was frequently present, with no evidence of significant gastritis in any case [63].

Several research groups did not find any correlation between NHPH infection and (the severity of) gastric lesions in dogs [60, 64–68]. However, Kubota-Aizawa et al. reported that infection of dogs with gastric NHPH, particularly *H. heilmannii* s.s., may be associated with more severe gastritis compared to dogs negative for NHPH. Furthermore, they suggested that gastric NHPH infection in dogs may be associated with a higher frequency of chronic diarrhea compared to dogs not infected with NHPH [69]. Experimental infection with *H. felis* in gnotobiotic Beagle dogs has been associated with histopathologic changes including lymphofollicular gastritis, with lymphoid nodules being most abundantly present in the fundus and body of the stomach [70]. Simpson et al. reported opposite findings after experimental infection with *H. felis* in specific-pathogen-free, *Helicobacter*-free Beagle dogs [71]. Taking into account the low density of colonization and limited duration of infection, they reported that *H. felis* is most likely not a gastric pathogen in dogs as they found no association between *H. felis* infection and the severity of gastritis in dogs. Mild gastric inflammation and lymphoid follicles were observed in both infected and uninfected dogs and the gastric secretory axis was not different in infected compared to uninfected dogs. In another study, Simpson et al. showed that the gastric function in dogs with a naturally acquired NHPH infection was not hampered and antimicrobial and antisecretory therapy administration caused suppression of the infection rather than eradication of the NHPH [72]. Again, several factors may underlie these contradictory findings, including differences in virulence of different species and strains of NHPH, the strain of the host, and the duration of infection.

As stated earlier, coevolution of a bacterium and its natural host generally leads to decreased pathogenicity. Dog- and cat-associated gastric NHPH coevolved with their host far before domestication of either cats or dogs. Therefore it is hypothesized that these NHPH cause mainly asymptomatic infections in cats or dogs and domestication has had little impact on the spread of these bacteria within the population of cats and dogs [1]. Furthermore, co-infections with multiple gastric NHPH species are commonly detected in dogs and cats. In that respect, dog- and cat-associated gastric NHPH can be regarded as sympatric (*“i.e. two bacterial species sharing the same niche and host who frequently encounter one another”*). Hence, extensive genetic exchange has been detected among these gastric NHPH, with this interspecies admixture being most evident in *H. heilmannii* s.s. and *H. bizzozeronii*. Also, the cat-associated *H. baculiformis* and dog-associated *H. cynogastricus* have been found to share a significant amount of DNA with *H. felis* and have therefore been defined as hybrids [1].

The prevalence of gastric NHPH in dogs and cats is found to be high, irrespective of clinical signs. The prevalence of gastric NHPH in clinically healthy dogs ranges between 67 and 86% and between 61 to 100% in dogs presented for investigation of chronic vomiting [9, 60, 64, 67, 68, 71, 73–76]. In naturally infected dogs, *H. bizzozeronii* was reported to be the most prevalent gastric NHPH [67, 77, 78]. Gastric NHPH in cats were detected in 41% to 100% of clinically healthy cats and were slightly more prevalent in cats presented for investigation of chronic vomiting [9, 59, 60, 75, 76, 79]. In cats, *H. felis* and *H. heilmannii* s.s. have been found to be the predominant NHPH infecting the gastric mucosa [61, 75].

Gastric NHPH have already been detected in the saliva and feces of dogs and cats, suggesting that transmission could happen via saliva and feces [64, 65, 80]. On the other hand, Craven et al*.* claimed that transmission via saliva is unlikely, based on the low frequency of NHPH detection in the saliva of dogs, compared to the frequent detection of NHPH in the gastric mucosa [81]. The exact mode of transmission still remains unclear.

## Zoonotic potential of gastric non-*Helicobacter pylori Helicobacter* species

It has been shown that *H. suis*, *H. felis*, *H. bizzozeronii*, *H. salomonis* and *H. heilmannii* s.s. have zoonotic potential and are potentially clinically relevant in humans [1]. The zoonotic potential of the other dog- and cat-associated gastric NHPH, *H. ailurogastricus*, *H. baculiformis* and *H. cynogastricus*, has not yet been established.

Prevalence rates of gastric NHPH infections in human patients undergoing a gastroscopy have been reported to vary between 0.2% and 6%, depending on the geographical area and the detection methods used [9, 82]. Populations where animals and humans live closely together and sanitary conditions are poor could be prone to an increased NHPH prevalence rate [64]. PCR-based studies investigating human gastric biopsy samples with histological evidence of presence of NHPH showed that *H. suis* had the highest prevalence (14 to 37%), followed by *H. salomonis* (21%), *H. felis* (15%), *H. heilmannii* s.s. (8%) and *H. bizzozeronii* (4%) [3, 75, 83]. These data predate the description of *H. ailurogastricus* and therefore no distinction between *H. heilmannii* s.s. and its closest relative, *H. ailurogastricus*, was made in these studies [29]. Co-infections with more than one NHPH or with *H. pylori* and NHPH have also been reported [75, 83–86].

Due to the very fastidious growth characteristics of gastric NHPH, their focal colonization pattern in the human stomach and the limited amount of gastric tissue that is usually available when a biopsy sample is taken from human patients, only very few gastric NHPH isolates from humans have been obtained worldwide. The first *H. bizzozeronii* strain was isolated from a human patient in Denmark in 2001 [87]. Later, this species was also isolated from a Finnish patient presenting with severe dyspeptic symptoms, including nausea and vomiting, and chronic active gastritis [88]. In Germany, *H. felis* was isolated in 2012 from the gastric mucosa of a 14-year-old girl presenting with persistent epigastric pain and episodes of vomiting without diarrhea, accompanied with mild chronic gastritis [89]. *Helicobacter felis* has also been isolated from a patient presenting with acute abdominal pain, nausea and vomiting of brown fluid, symptoms that appeared to be related to severe acute gastritis [90]. Only recently, in 2020, Rimbara et al. succeeded in obtaining pure cultures of *H. suis* strains from three human patients with gastric diseases, including gastric MALT lymphoma [47, 91]. Isolation and cultivation of other gastric NHPH from human patients with gastric diseases has not yet been described.

Transmission of gastric NHPH to humans is generally suggested to happen through direct or indirect contact with dogs, cats or pigs, and potentially via consumption of contaminated water [9, 83, 92–99]. For *H. suis*, an additional means of transmission may include consumption of contaminated pork [100]. These means of direct and indirect transmission are further validated by reports of molecular analyses proving the great genetic similarity of gastric NHPH strains present in humans and these animal hosts [8, 47, 61, 93, 94]. Furthermore, since laboratory mice are easily colonized by most gastric NHPH, the role of wild mice as a vector for the transmission of gastric NHPH should also be considered. Nunes et al. raise awareness for the potential role of wild boars and other wild animal species as a reservoir for *H. suis* and possible source of infection for humans [46, 101]. Although, several earlier reports revealed that *H. suis* is absent in the majority of wild boar populations, which is in accordance with the fact that the host jump of *H. suis* from non-human primates to pigs occurred rather recently [8]. Therefore, this route of transmission needs further investigation. To date, no cases of human-to-human transmission have been reported [8].

Infection with gastric NHPH in humans has been associated with chronic gastritis, peptic and duodenal ulcer disease and low-grade MALT lymphoma of the stomach [7, 9, 82, 102–106]. Occasionally, cases of acute gastric mucosal lesions have also been reported [90, 107]. Since the risk of developing gastric MALT lymphoma is higher with NHPH than with *H. pylori* [108] the association between gastric NHPH and gastric MALT lymphoma has been most clearly described in literature. In addition, many studies have been performed on eradication of gastric NHPH as a potential cure for MALT lymphoma. On the other hand, the severity of lesions associated with chronic gastritis caused by gastric NHPH has been reported to be lower compared to *H. pylori* [108, 109].

Several research groups have recently investigated the clinical relevance of the potentially zoonotic gastric NHPH in large cohorts of patients. Among 236 Japanese gastric disease patients who tested negative for *H. pylori* based on a rapid urease test, 49 (20.8%) tested positive for NHPH using PCR and immunohistochemical methods. *Helicobacter suis* was detected in 20 patients, *H. heilmannii* s.s./*H. ailurogastricus* in 7 patients and the other 22 species remained unidentified. Gastric diseases within this group of patients included nodular gastritis, gastric MALT lymphoma, chronic gastritis and gastroduodenal ulcers, with no significant association between the *Helicobacter* species detected and the types of disease diagnosed. Standard eradication therapy was administered to 45 of these NHPH positive patients. This led to successful eradication of the infectious agent in all cases based on PCR results and improvement of endoscopic findings in most cases, except for some MALT lymphoma cases [82]. Furthermore, out of 3847 Japanese patients who underwent biopsy of the gastric mucosa between 2010 and 2020, 50 patients (1.30%) were diagnosed with NHPH gastritis based on microscopic findings after Giemsa staining. PCR analysis detected mainly *H. suis* (26/28) (2/28 were *H. heilmannii* s.s./*H. ailurogastricus*) and pathology was described as “crack-like mucosa” and nodular gastritis in the gastric antrum and regular arrangement of collecting venules in the gastric corpus [110]. In Turkey, a study including 110 children with gastric complaints revealed the presence of *H. suis* infection in two cases and *H. heilmannii* s.s./*H. ailurogastricus* in one case by performing PCR analysis. The former presented with active chronic gastritis without macroscopic lesions, while the latter presented with hyperemia without presence of gastric inflammation. Also, a mixed infection with *H. suis* and *H. pylori* was detected in one patient who presented with gastric ulceration in combination with moderate, active chronic gastritis [111]. Similar studies in western hospitals are currently lacking, but such studies are needed to establish the clinical relevance of these potentially important zoonotic NHPH and potential eradication programs.

Also of clinical interest, *H. pylori* infection has been described to play a role in the development and progression of central nervous system (CNS) disorders being linked, for example, to an increased risk of all-cause dementia [112] and to the onset of idiopathic parkinsonism (IP) and its motor symptoms [113–115]. Recent findings also revealed possible associations between *H. suis* infection and CNS disorders, including IP and Alzheimer’s disease (AD). In gastric biopsies from IP patients, a significantly higher presence of *H. suis* DNA (27%) was detected compared to control patients without evidence of clinical IP symptoms (2%) [10]. In addition, a blood sample from a patient affected by IP and AD tested positive for *H. suis* DNA. Following eradication therapy, both gastric and neurological symptoms improved substantially [10]. Recently, *H. suis* infection in IP patients was also associated with higher mortality [116]. It is not yet clear how a gastric *H. suis* infection might impact the CNS [114]. One hypothesis starts from gastric inflammation induced by *H. suis*. This is associated with loss of the gastrointestinal barrier function, causing a leaky gut with leakage of TLR4 ligands (and other molecules) into the blood. This might affect the blood-cerebrospinal fluid barrier and induce microgliosis, resulting in cognitive decline. Another pathway assumes a possible relationship with production of gamma-glutamyl transpeptidase and urease by *H. suis* which might result in a depletion of glutamine, a precursor for several neurotransmitters, and continuous slightly higher levels of ammonia, respectively. Changes in the gastrointestinal microbiome associated with a *H. suis* infection, might also impact brain homeostasis. Similar to how *H. suis* has already been associated with changes of the microbiome of the *pars oesophagea* of the porcine stomach, gastric NHPH have indeed been associated with shifts in structure and function of the gastric microbiome in human patients too [117]. The exact relevance in the pathogenesis of gastric diseases and the development of CNS disorders remains to be further investigated.

The efficacy of administration of standard *H. pylori* eradication therapy, typically consisting of a proton pump inhibitor and 2 or 3 antibiotics, in order to eradicate the gastric NHPH and achieve remission of the associated gastric pathology, is currently being investigated [99, 118–121]. In a patient diagnosed with *H. suis* infection and chronic gastritis, 10 days of standard triple therapy (esomeprazole, metronidazole and amoxicillin) led to *H. suis* eradication and regression of the gastric mucosal lesion upon esophagogastroduodenoscopy (EGD) three months post therapy. Also, there was no recurrence of similar lesions upon EGD within two years after administration of the eradication therapy [118]. Another case report demonstrated that, three months post standard triple eradication therapy in a patient with asymptomatic *H. suis* gastric MALT lymphoma, the bacterium was no longer detected, the erosive mucosal lesions improved, small lymphocyte infiltration decreased and lymphoepithelial lesions had disappeared. However, lymphoid nodules were still present. After nine months, no lymphoid infiltrations could be detected and complete remission was achieved [119]. Furthermore, a complete response rate to eradication therapy was achieved in 4 cases of *H. suis* positive gastric MALT lymphoma with nodular gastritis-like appearance, where both the infection and gastric disease were eradicated. Standard triple eradication therapy in a *H. heilmannii* s.s. infected patient diagnosed with multiple gastric ulcers also led to ulcer improvement and *H. heilmannii* s.s. eradication [99]. Other research groups reported similar results [103, 110, 122, 123] and confirmed earlier animal studies [124]. These findings suggest a definite causal relationship between gastric NHPH infection and the development of gastric disease [125].

Antimicrobial susceptibility tests have revealed that acquired antimicrobial resistance occasionally occurs in several gastric NHPH, including *H. suis* isolates from pigs and macaques [126], feline *H. heilmannii* s.s. isolates [31] and feline *H. felis* isolates [127]. The minimum inhibitory concentration (MIC) of amoxicillin for a *H. suis* isolate obtained from a human patient (16 mg/L) was approximately 10 times higher than that of three other isolates from humans (1 mg/L) which might be due to several mutations in the penicillin binding proteins PBP2 and FtsI (PBP3) [47]. Presence of acquired resistance in some NHPH isolates might complicate treatment as already described for *H. pylori*. Determinations of MIC indicated that porcine *H. suis* strains are intrinsically less sensitive to aminopenicillins than non-human primate *H. suis* strains and other gastric *Helicobacter* species. *Helicobacter suis* strains also seem to be intrinsically less susceptible to metronidazole than *H. pylori* strains. Although aminopenicillins and metronidazole are often used for treatment of human patients infected with gastric *Helicobacter* species, the possible therapeutic significance of these findings is not clear [128].

## Rodent models in research for gastric non-*Helicobacter pylori Helicobacter* species

Rodent models (particularly mice and gerbils) are frequently used to understand the basic principles of gastric NHPH infection as well as for translational research. This ranges from patho- and immunogenicity studies to the search for potential curative and preventive therapies. For instance, experimental infection in mice with a *H. suis* strain originally isolated from pigs has been suggested to elicit a predominant Th2/Th17 response [129, 130], where a Th2 response may be associated with B cell accumulation leading to the development of gastric MALT lymphoma [131]. In another study, inoculation of mice and Mongolian gerbils with *H. suis* strains isolated from stomachs of non-human primates and pigs has been shown to induce a Th17 response and elevated CXCL13 levels accompanied by severe gastric inflammation. The immune response evoked was shown to depend on the experimental host rather than the original host to which the strain belongs [132]. Upregulation of CXCL13 levels has also been observed in *H. suis* infected pigs [44, 133]. Since CXCL13 is a chemokine which attracts B cells, this pathway is hypothesized to be involved in the development of gastric MALT lymphoma associated with gastric NHPH infection. Furthermore, mice experimentally infected with *H. felis* have long been used as a model for human *H. pylori* infection [134].

In order to screen for potential antibiotic and other treatment regimens, several mouse models have been developed [124, 135–137]. Guided by the mindset that administration of certain *Lactobacillus* strains might lead to reduced gastric NHPH colonization, administration of the *Lacobacillus gasseri* strain SBT2055 in a C57BL/6 J mouse model infected with *H. suis*, effectively suppressed the progression of gastric MALT lymphoma [138]. Vaccination as a mode of prophylactic immunization against NHPH, including *H. felis* and *H. suis*, has been tested in mouse models as well, but no formulation has provided complete protection yet. Major side effects, including post-vaccination gastritis and increased mortality rates, have been reported and have hampered this research domain [139–141]. However, promising results were obtained in a study which proved inhibition of the development of MALT lymphomas after immunization based on *H. felis* whole cell sonicate in a mouse model challenged with *H. felis* [142]. Completely unexpectedly, it was recently discovered that experimental infection with *H. suis* in a 6-OHDA Parkinson’s disease mouse model had (partial) neuroprotective effects on Parkinson’s disease pathology, mediated by gene expression changes in brain tissue related to reduction of oxidative stress [143].

Although rodent models are indispensable in gaining novel insights into pathogenicity and possible cure, significance in the relevant host, and thus translatability, must always be taken into account.

## Virulence mechanisms of gastric non-*Helicobacter pylori Helicobacter* species

An overview of the virulence factors and their roles in pathogenesis is presented in De Witte et al. [56]. The most important virulence factors of gastric NHPH are briefly summarized below.

Gastric helicobacters as a group share several virulence mechanisms, although some virulence factors are *Helicobacter* species- and even strain-dependent. They are mainly involved in colonization, induction of gastric pathologies and host immune evasion.

Gastric helicobacters are capable to survive the extremely acidic conditions of the stomach lumen and, at the same time, they can penetrate the outer mucus layer covering the gastric mucosa in order to reach their ideal niche which has an external pH of 5 to 6.

Characteristically, all gastric helicobacters produce the enzyme urease, containing subunits UreA and UreB which are assembled into a catalytically active, nickel-containing dodecamer via the actions of the accessory proteins UreE, UreF, UreG and UreH [144, 145]. An inner membrane protein, UreI, forms a proton-gated urea channel in order to upregulate urease activity under acidic conditions and allow rapid entry of urea into the cytoplasm [146]. Urease hydrolyzes urea into carbon dioxide and ammonia, hereby neutralizing the gastric acid and creating a pH neutral microenvironment surrounding the bacterium [9]. Urease activity and UreI have both been proven to be essential in the pathogenesis of *H. pylori*, as colonization in animal models of infection is hampered in case either one of these is lacking [146–148].

In order to allow the gastric helicobacters to penetrate the outer mucus layer and move towards the gastric mucosal surface where they naturally reside, motility of the bacteria is essential. To this end, gastric NHPH make use of their helical shape and the presence of bipolar flagella [9, 149, 150]. The ideal niche for NHPH is located deep in the gastric pits, as opposed to *H. pylori*, which is usually detected at the surface epithelium [9]. Furthermore, gastric helicobacters possess genes encoding proteins involved in chemotactic behavior. As such they are able to sense the surrounding pH and move away from low pH regions towards more neutral regions [9]. *Helicobacter pylori* has been found to prefer the colonization of injured epithelium as it may possess high amounts of favorable nutrients [151] and sense the amount of bacteria in close proximity in order to spread out and avoid shortage of resources [152].

Adherence and binding to epithelial cells is an important step in the colonization of the gastric mucosa by helicobacters. To this end, several mucins and glycolipids expressed on the apical surface of gastric mucosal epithelial cells play a crucial role. Mucins are long and filamentous glycoproteins and therefore, an ideal first point of interaction between the host tissue and helicobacters [153, 154]. In BALB/c mice experimentally infected with *H. heilmannii* s.s., expression of the secreted mucin Muc6 and the transmembrane mucin Muc13 was upregulated in the first 9 weeks post-infection [155]. A positive correlation was found between the number of *H. heilmannii* s.s. bacteria and the increased expression of Muc6 in the antrum and Muc13 in the fundus, suggesting the importance of these mucins in *Helicobacter* colonization. Upregulation of Muc13 expression remained until 52 weeks post-infection, confirming earlier suggestions about its potential involvement in promoting the transition from chronic inflammation to cancer [156]. Mucins, such as the cell surface mucin MUC1 to which *H. pylori* is able to bind, have also been shown to serve as a releasable decoy in order to inhibit colonization by mucin-binding bacteria, and have been shown to create steric hindrance in order to avoid adhesion of non-mucin binding bacteria to the cell surface [153]. Also, *H. suis* infection has been shown to lead to a decrease in glycan structures to which *H. suis* is able to bind in the pig gastric mucus niche, a decrease in the *H. suis*-binding ability of human mucins, and to alter the porcine and human mucin composition in a way that the growth-inhibiting effect of mucins gives way to a growth-enhancing effect. This is thought to lead to a more stable and inhabitable niche for *H. suis*, likely supporting long-term colonization and outcome of infection [157]. Furthermore, *H. suis* has been shown to bind to both human and pig mucins and glycolipids via two modes of adhesion. One mode of adhesion is to glycans terminating with Galβ3GlcNAc (eg. lactotetraosylceramide present in pig stomach) at both neutral and acidic pH and the other is to negatively charged structures at acidic pH [158]. Since the glycosylation profile of gastric mucosa is dependent on the animal species, glycan receptors involved in the binding by different *Helicobacter* species have been found to differ, as part of host adaptation. Essentially, different gastric NHPH dispose of different adhesion mechanisms mediated by proteins with alternative receptor specificity. In the canine gastric mucosa, NHPH have been suggested to possibly bind via terminal α-GalNAc and type 2 Lewis antigens, and *H. heilmannii* s.s. has been shown to adhere the most [159]. The significance of these findings in the physiopathology of NHPH infection in dogs remains to be further investigated.

The genome of gastric helicobacters encodes a large set of outer membrane proteins (OMPs) involved in colonization and virulence. According to comparative genomic studies, gastric NHPH lack all known adhesins discovered in *H. pylori* so far [29, 160–162]. However, they share few homologs of the Hor and Hom family [160, 162] and in *H. heilmannii* s.s., HofE and HofF have been identified as adhesins with an important role in IL-1β induced gastric MUC13 expression [163]. Proteomic and phylogenetic analysis of the OMPs of gastric NHPH identified the Hof proteins as a *Helicobacter*-specific OMP family, found widespread amongst all gastric *Helicobacter* species, with a putative function in adhesion (Family 33) [162]. This study also discovered a *Helicobacter*-specific SfpA/LpxR OMP (Family 36) important in immune evasion, possibly via lipopolysaccharide modulation, and a *Helicobacter*-specific vacuolating cytotoxin A (VacA)-like cytotoxin family with a function in colonization capacity (Family X3) in gastric helicobacters. Presence of an outer membrane factor (OMF) (Family 42) and Imp/OstA (Family 14), both important in antimicrobial resistance, TonB-dependent OMPs with a function in metal and vitamin-uptake (Family 3 and Family X2), and an outer membrane phospholipase (OMPLA) (Family 38) involved in colonization capacity have also been identified in most gastric *Helicobacter* species. So, it is known that *Helicobacter*-, species- and even site-specific members of OMPs/OMP families exist, but their significance in virulence and colonization remains to be further explored [162].

Regarding virulence factors associated with induction of gastric pathologies, both cytotoxin-associated gene pathogenicity island (cagPAI) and VacA, which are two major cytotoxic virulence factors in *H. pylori* [5], are not present in any of the gastric NHPH discussed in this review.

Gamma-glutamyltranspeptidase (GGT) is an epithelial cell death inducing virulence factor present in each gastric *Helicobacter* species in a highly conserved manner. The metabolic function of GGT for gastric NHPH is to break down extracellular glutathione (GSH) and glutamine in order to produce glutamate, which is incorporated intracellularly into the tricarboxylic acid cycle. This glutamate-generating metabolism is essential for survival and growth of gastric helicobacters [164]. Gamma-glutamyltranspeptidase as a virulence factor has been identified as a direct causal factor for *Helicobacter*-induced gastric disorders. Unraveling of the underlying molecular mechanism has shown that degradation of extracellular reduced GSH by GGT generates GSH degradation products which increase the extracellular concentrations of reactive oxygen species (ROS) in a cell-independent manner, resulting in oxidative cell damage on different levels, including cellular lipid peroxidation. This oxidative stress induced cell damage ultimately leads to different types of cell death of gastric epithelial cells, with apoptosis in case of low extracellular ROS concentrations and oncosis/necrosis in case of high extracellular ROS concentrations [165].

Several other possible virulence factors have been determined to be encoded for in the genome of gastric NHPH based on genome sequence analyses of *H. suis* [160], *H. felis* [166], *H. bizzozeronii* [161] and *H. heilmannii* s.s. [167], but their exact significance in the development of gastric pathologies associated with NHPH infections remains unclear.

In addition to aforementioned OMPs and other virulence factors involved in host immune evasion for persistence of infection, gastric helicobacters also possess the ability to influence dendritic cell maturation and cytokine production. In pigs, induction of semi-maturation of monocyte-derived dendritic cells by *H. suis* has been observed, eliciting expansion of regulatory T cells [133]. Via this regulatory T cell response, a tolerogenic phenotype is induced, thereby stimulating chronic colonization [133, 168]. Since *H. pylori* urease has been found to bind CD47, thereby impeding the folding and transport of the MHCII molecules [169], and since this is a highly conserved virulence factor among gastric helicobacters, urease is hypothesized to play a role in host immune evasion. Furthermore, GGT and L-asparaginase II have been found to inhibit T cell proliferation, further promoting the tolerogenic phenotype [170, 171].

In *H. ailurogastricus*, several homologs encoding *H. pylori* virulence and colonization factors previously identified in *H. heilmannii* could not be retrieved and it was found to have a lower binding capacity for gastric epithelial cells in vitro. It is therefore suggested to be a low-virulence gastric NHPH [29].

Besides disparities in virulence among the gastric NHPH, strain-specific differences have also been identified. Rimbara et al. observed differences in the progression of gastric mucosal metaplasia in mice inoculated with different *H. suis* strains isolated from human patients, indicating variation in virulence between *H. suis* isolates, as has frequently been reported for *H. pylori* [47]. In the accessory genome of a more virulent *H. suis* strain, many genes were detected which were absent in a less virulent strain, including type 4 secretion system-associated genes located in plasticity zones. Further studies are necessary to unravel the significance of this finding.

## Conclusions

Besides *H. pylori*, several other gastric helicobacters appear to be clinically relevant in humans. At least four dog-, cat- and pig-associated gastric NHPH have been detected in humans and have been proven to be a causal factor in gastric disease. Clinical and preclinical research has provided evidence regarding pathogenic mechanisms and possible treatment strategies. However, a lot remains to be investigated, including true prevalence rates, exact modes of transmission and other zoonotic aspects, and molecular pathways underlying disease development and progression. Further elucidation of these research questions will require large patient cohort studies incorporating standardized detection methods. Also, the optimization of in vitro isolation methods for these extremely fastidious gastric NHPH could be of great value for a better understanding of the pathogenic mechanisms and developing potential treatment and prevention strategies. To date, 8 gastric NHPH which are commonly present in dogs, cats and pigs have been fully characterized. The clinical significance of *H. suis* in pigs has been established, whereas the clinical significance of cat- and dog-associated gastric NHPH in their natural host remains controversial or yet to be explored. However, evidence regarding coevolution suggests that this may be low.
